# Prevalence and Correlates of Physical Inactivity during Leisure-Time and Commuting among Beneficiaries of Government Welfare Assistance in Poland

**DOI:** 10.3390/ijerph14101126

**Published:** 2017-09-26

**Authors:** Dorota Kaleta, Sylwia Kalucka, Franciszek Szatko, Teresa Makowiec-Dąbrowska

**Affiliations:** 1 Department of Hygiene and Epidemiology, Medical University of Lodz, 90-647 Lodz, Poland; sylwia.kalucka@umed.lodz.pl (S.K.); franciszek.szatko@umed.lodz.pl (F.S.); 2 Department of Work Physiology and Ergonomics, Nofer Institute of Occupational Medicine, 91-348 Lodz, Poland; tmd@imp.lodz.pl

**Keywords:** physical activity, commuting, social, walking, bicycling, leisure, correlates, disadvantaged groups, low socio-economic status population

## Abstract

Physical activity (PA) has well-documented health benefits helping to prevent development of non-communicable diseases. The aim of the study was to examine the prevalence and factors associated with physical inactivity during leisure-time (LTPA) and commuting (CPA) among adult social assistance beneficiaries in Piotrkowski district. The studied sample consisted of 1817 respondents. Over 73% of the study population did not meet the recommended levels of LTPA. Fifty two % of the respondents had none leisure-time physical activity and 21.5% exercised occasionally. Main reasons for not taking up LTPA included: high general physical activity (36.4%), lack of time (28.1%), no willingness to exercise (25.4%). Close to 82% of the surveyed population did not practice commuting physical activity (CPA). The men had higher risk for inactivity during LTPA compared to the women (OR = 1.35; 95% CI: 1.11–1.65; *p* ≤ 0.05). Higher odds of CPA inactivity were associated with unemployment, moderate and heavy drinking and having a number of health problems. The prevalence of physical inactivity among the social assistance recipients is much higher than it is in the general population. Promotion of an active lifestyle should take into consideration substantial differences between the general population and disadvantaged individuals and their various needs.

## 1. Introduction

Physical activity (PA) is a low-cost, non-pharmacological, but well-documented and the most effective way to maintain good health, self-rated health and prevent development of non-communicable diseases (e.g., cardiovascular diseases) [1]. Regular physical activity is associated with a number of health benefits, including: a reduced risk of premature mortality (mainly because of cardiovascular diseases), obesity, diabetes [2,3] as well as some kinds of cancer (e.g., breast cancer [4,5], colorectal cancer [6]) and depression [7]. Despite the well-known benefits of physical activity, there is a worldwide trend towards lesser and lesser total daily physical activity. Nowadays, it is estimated that globally, approximately, one third of adult population do not achieve the recommended levels of physical activity [8]. In Europe, the studies reveal that more than one third of adults are insufficiently active [9]. Recent data from the European Union countries (EU) indicate that six in every 10 people above 15 years of age never or seldom exercise or play a sport, and more than one half of them never or seldom engage in any other kinds of physical activity, such as: cycling, dancing or gardening [8]. At the same time, a high proportion of adults in Europe spend more than four hours a day sitting, which could be a contributing factor to a sedentary lifestyle [9]. The level of physical activity is also not satisfactory among Polish population [10]. In 2011 less than one half of adult working population had PA lasting at least 30 min on most days of a week and less than one third of the studying population commuted actively [11]. As a consequence, physical inactivity has become a leading risk factor for ill health. Physical inactivity is estimated as the fourth leading risk factor for global mortality, which is responsible for about 3.2 million deaths yearly [12]. In the WHO European Region about 1 million deaths (about 10% of the total) and 8.3 million disability-adjusted life years lost per year are attributable to physical inactivity. Moreover, physical inactivity is estimated to cause 5% of the burden of coronary heart disease, 7% of type 2 diabetes, 9% of breast cancer and 10% of colon cancer [3]. Therefore, the European Union, the United States Department of Health and Human Services and the World Health Organization promote a healthy active lifestyle and recommend undertaking regular physical activity [13,14,15]. It has been observed that the prevalence and correlates of LTPA depend on a country and on an ethnic group, and this aspect has been studied most widely in Western countries, mostly examining urban populations [16]. Research on the correlates of physical activity among disadvantaged subgroups and populations is relatively scarce, especially in rural Poland.

In order to develop effective interventions and to control a physically inactive population, we need to find out and understand which factors influence people’s decision to practice physical activity or not to do it. There are many determinants associated with physically inactive adults. Physical activity constitutes a combination of behaviors, which are determined by interaction of a large number of factors. These determinants include: a social, personal, environmental setting and a kind of physical activity. All these factors are dependent on an individual group (e.g., disadvantaged), ethnic subgroup and whole populations. For instance, a study conducted in 28 European countries, has shown that a low physical activity was correlated with: being unemployed, being a student, being a retired person and having a higher household income [17]. Most studies examining the impact that a residential location has on physical activity, have shown that the recommended physical activity guidelines are less likely to be met by residents of rural areas when compared to the residents of urban ones. Differences between residents of urban and rural areas have been highlighted by several studies. Inter alia, studies by Parks, Housemann, and Brownson have reported significant differences in the importance of venues to exercise with respect to physical activity behavior [18]. Namely, urban adults found it important to have access to parks, walking trails and exercise equipment. At the very same time it was access to neighborhood streets for activity and an indoor gym that were more important for rural adults.

Moreover, comparison between the rural environment and urban one, reveals different barriers to being physically active. According to the existing data, the most important barriers for rural women to do physical exercises are: caregiving duties and the remoteness, while in the case of urban women it is lack of time [19]. Rural women do not pay attention to pavements, streetlights, high crime, access to park or facilities in their area, as opposed to women who live in the city, where these are essential reasons for continuing PA [20]. Rural residents with a lower income, low level of education, greater body mass indices are less likely to meet physical activity recommendations than residents of urban areas [18,19,20]. On the other hand, some studies have noted that the prevalence and correlates of different types of physical activity differ across countries, ethnic groups, cultures and genders. In economically advanced and rapidly developing countries people spend more and more time on occupational activities at the expense of leisure time, which they should spend relaxing and doing physical activity. Long working hours (mainly sitting at work) and psycho-social work demands have a negative impact on leisure time physical activity (LTPA). People with a low level of occupational physical activity (OPA) are at risk of inactivity and have developed obesogenic behaviors (e.g., lack of physical activity, sedentariness on the job) [21]. Therefore, prevention of development of non-communicable diseases cannot be limited only to LTPA, but it should also include another type of physical activity, i.e., commuting (CPA—walking or biking to work). Some participants may experience barriers towards regular commuting physical activity. One of them is associated with the presence of vehicles for both sexes in a household [22]. In addition, even high educational and occupational status urban women who in general exercise more, have negative predictors of total and leisure-related PA because of major barriers to being physically active, such as: weather, tradition, lack of facilities and time [23]. Sometimes, because of so diverse socio-economic factors (e.g., religion, tradition, customs) it is difficult to recommend one particular type of PA. Even though all positive effects of PA are well known, it is very difficult to maintain a moderate to vigorous level of physical activity in the general population for a long time. It is even more problematic in the case of disadvantaged people, who on a daily basis have numerous difficulties (not only economic) [24]. Most studies indicate that PA during leisure-time is more common and frequent in high socio-economic groups when compared to low socio-economic ones. However, these socio-economic differences are not observed in transport PA in European adults [25] and some Asia-Pacific countries [26]. Generally, the prevalence of physical inactivity in some countries is associated with low education [27,28,29], elderly age [28], low socio-economic status (SES) [28] and more health problems [10,29]. But there are some countries where for example age is not a problem and a regular physical activity increases with it and people live the longest in the world [26,30]. Total physical inactivity is negatively correlated with unfavorable neighborhood indicating that unfavorable neighborhood-social culture and religion conditions [29], lack of support groups —discourages PA [31,32]. On the other hand, in some populations good access to exercise facilities and a friendly, safe neighborhood environment can determine a specific type of physical activity and significantly increase the level of each physical activity in all ages (even elderly or disadvantaged) [30,33]. Different populations have diverse needs and challenges with regard to the promotion of physical activity. This needs to be taken into account by policy-makers. Studies on many aspects of physical activity have been published but detailed data on the prevalence of physical activity and commuting PA among disadvantaged people are scarce. Disadvantaged groups remain the most heterogeneous group with differentiated needs and problems (health, social, functional status and other). Furthermore, little is known about the factors that may strongly influence physical inactivity in the socially disadvantaged people. Knowledge is needed for present and future generations, because a sedentary lifestyle in parents is a negative pattern for their children. Children, whose parents were physically inactive, had 147% higher odds of copying their parents’ behavior than children whose both parents were physically active [34]. Therefore, it is important to understand the disadvantaged group and the correlates of PA participation. Up till now, no data have been published that would present both types of physical activity (LTPA and CPA) in disadvantaged groups in Poland.

The primary purpose of this study was to examine the prevalence of physical inactivity during leisure-time (LTPA) and commuting physical activity (CPA) among adult social assistance beneficiaries, and to analyze factors associated with the lack of physical activity (in both domains) in the study population.

## 2. Materials and Methods

### 2.1. Characteristics of the Examined Region

Piotrkowski district (Piotrków Trybnalski, Poland) was selected to perform studies and implement interventions among beneficiaries of government welfare assistance. Piotrkowski district is situated in the center of Poland and belongs to Lodzkie voivodeship (Lodz Province, Poland). The district is located on the area of upland. Flatland prevails, which favors agriculture and animal breeding. One fourth of the district is forest, which constitutes the most serious component of natural resources in this region. Piotrkowski district is rich in sand and gravel deposits that are used mainly in the construction industry. According to the data of 31 December 2012 on the premises of Piotrkowski district there were in total 5199 administrative entities (182 in public sector and 5017 in the private one). Based on the REGON register (National Official Register of Entities of the National Economy) during this period, the prevailing entities functioned in the field of: wholesale and retail, car repair (1551), construction (727), agriculture, forestry, hunting and fishing (307). In terms of surface, small and medium farms (up to 20 ha of arable land) dominate, belonging to the mechanized category to an average or elementary extent. Agricultural lands belong mainly to individual farmers. On the premises of the district farms specialized in the production of different grains prevail. Most seldom business existing on the premises of Piotrkowski is related to the real estate industry, culture, entertainment and recreation (92) [35,36].

Piotrkowski district was selected based on the analysis written by the United Nations Development Program (UNDP) because of a few reasons. Firstly, in 2013 in Piotrkowski district there were about 9% of residents who required support of social assistance institutions [35]. Another reason was the fact that this region was identified as the 11th among all 314 rural districts with the lowest indicators of social development in Poland [36]. The indicators of social development were based on the Local Human Development Index (LHDI). The LHDI index including three indicators: Health Index, Education Index and Welfare Index amounts to 25.97, with Health Index (HI) equal 26.50. However, in Lodzkie vioivodeship the LHDI index amounted to 39.28 with HI-31.48, respectively [37]. The poverty threshold, as adopted by the social assistance institutions, was determined as the income lower than specific poverty lines as identified in the Social Assistance Act [35]. This approach to the poverty line is binding upon all the district level social assistance institutions, where within the group of 11,867 social assistance beneficiaries there were 4336 families [35]. The further reason was, that Piotrkowski district is one of 156 Polish districts where overall mortality rate is the highest and mortality arises from the top five causes of death [38,39].

Taking this into consideration Polish government along with the Norwegian Directorate of Health Affairs have implemented Project PL-13 “Reducing social inequalities in health” [38]. This project was funded by the Polish state and the Norwegian Financial Mechanism 2009–2014 (www.eeagrants.org). During this period, 156 Polish districts with the highest overall mortality rates took part in it. It aimed mainly at reduction of inequalities in health among the residents of Piotkowski district through an initiative known as “Your heart is your life”. There were a number of actions (e.g., health promotion) targeting the socio-economically disadvantaged population. In this study, the socio-economically disadvantaged people are defined as individuals who have minimum income of no more than 634 PLN (148 Euro) for a single person monthly, and 514 PLN (120 Euro) for a family member monthly [36]. At the same time average income was 1340 PLN (313 Euro) for a single person monthly. In Poland income of an individual citizen is assessed based on PIT (individual income tax) that is obligatorily submitted each year to the tax office. During the study income of the participants was verified based on the available financial sources (PIT). There was no way to check if the partcipants have other, additional sources of income. Another form of assessing the low level of life was a question addressed to the participants and included in the questionnaire—subjective assessmen of monthly income.

### 2.2. Characteristics of the Study Sample

According to the Central Statistical Office, in 2013 there were 91,618 residents (including 45,223 men and 46,395 women) living on the premises of Piotrkowski district [35]. More than 90% of those residents lived in rural areas. Then there was a group of 11,867 social assistance beneficiaries, including 3636 adult residents aged 18–59 years.

A cross-sectional study was carried out among the residents of Piotrkowski district who received aid from the local social assistance organizations. The list of the recipients was provided by the local government. The study covered all the individuals registered in the local government welfare assistance institutions. The socially disadvantaged people agreed in writing to take part in the study and they fulfilled the inclusion criteria. The study was approved by the Bioethics Committee of the Medical University of Lodz in Poland (No. is RNN/243/15/KE). Data sources were derived from representative population surveys of individuals aged between 18 and 59 years (both males and females) who benefited from the local social assistance. The surveys were conducted between October 2014 and November 2014 among those beneficiaries who at the time of the study resided in Piotrkowski district. Data collected from the participants concerned the month preceding the study. At that time the climate was moderate and it was possible to be physically active.

### 2.3. Characteristics of the Participant’s Survey

The study was carried out by the use of a questionnaire, which was adopted from the Multi-Center National Population Health Examination Survey (WOBASZ) [38]. In the questionnaire closed questions prevailed. They gave the participants a choice of answers only within a particular category. Qualitative data prevailed in the questionnaire and that was its another feature. Only singular data were quantitative (e.g., BMI, number of smoked cigarettes). The questionnaire was completed by qualified interviewers at the respondents’ places of residence during face-to-face interviews. Actual performance of the surveys was preceded by several training meetings for all the survey staff, and then verified by a pre-test. Data collection was coordinated and supervised by a principal investigator, study coordinator and two field supervisors.

The questions included in the survey form covered a number of important issues, which were determined by four purposes of the study.

#### 2.3.1. Basic Demographic Data

Firstly, basic demographic data have been collected. They included: gender (female and male), age (in years, divided into several groups), education (primary, vocational, secondary, high) and employment status (permanent job, temporary job, disabled or retired, student, unemployed). The questionnaire also included questions on the subjective assessment of: monthly income, living conditions, cohabitation with somebody and having children. Monthly income was assessed according to five categories: sufficient to cover all living needs plus he/she may save a certain amount, sufficient to cover all living needs, sufficient to cover basic needs only, not sufficient to cover even basic needs, declined, don’t know. Subjective assessment of living conditions was classified as: fair, rather fair, neither fair nor poor, rather poor and poor. Information connected with cohabitation was as follows: living alone, living with a partner and/or a family. In the case of having children—children older than 15 years old, the participants answered ‘yes’ or ‘no’.

#### 2.3.2. Health Status & Health’ Behaviors

Second group of questions concerned health status and behaviors. The subjective health condition was rated as follows: fair, rather fair, neither fair nor poor, rather poor, poor. The number of health problems was divided into: none, 1–3 health problems, 4–6 health problems and 7 and more. The respondents’ weight was classified as obesity (Body Mass Index—BMI equal and over 30) and others in the case of those whose BMI was beyond 30. In the case of cigarette smoking the categories were as follows: a past smoker, an active smoker and never smoked. The participants’ answers to the question on alcohol consumption were interpreted as follows: not drinking at all, a moderate and a heavy drinker.

#### 2.3.3. Physical Activity & Reasons of Physical Inactivity

Finally, there were questions related to physical activity. There were two categories of physical activity: leisure-time physical activity (LTPA) and commuting physical activity (CPA). In the assessment of LTPA, the participants were asked whether they regularly practiced any physical activities (e.g., walking, jogging, cycling, swimming, gymnastics, gardening, except for active commuting) that would last at least 30 min. Those who did were asked to recall the frequency of such activities. The frequency of the respondents’ PA weekly was divided into 4 possible answers, such as: none, occasionally, 2 to 3 days per week, and 4–7 days per week. The individuals who did not declare doing any physical exercises in their leisure time were defined as ‘physically inactive’ and were asked about possible reasons for their inactivity. Other information collected in the questionnaire concerned reasons for not taking up any physical activity, for example: lack of time, no willingness to exercise (I have no such a need), bad health condition (a disease, disability), high general physical activity (physical work, a house, a garden plot etc.), lack of money or no access to suitable facilities (a gym, playing field etc.). In the assessment of CPA, the participants were asked about the daily commuting return journey, which was divided into four categories: by using motor vehicles (0 min of walking or cycling), by walking or cycling for 1–14 min, by walking or cycling for 15–29 min, and by walking or cycling for 30 min.The research covered all those who met the inclusion criteria and agreed in writing to participate in the study. A written informed consent was obtained from all the study participants, while the Bioethics Committee of the Medical University in Lodz positively reviewed the project (Project Identification Code: RNN/243/15/KE). The dataset can be found in the Supplementary Materials (SM).

Detailed characteristics of Piotrkowski District, the program assumptions and the methodology have all been published elsewhere [40].

### 2.4. Characteristics of the Statistical Analyses

The chi-square test was used to statistically analyze the four subgroups of the participants in the following age ranges: 18–29, 30–39, 40–49 and 50–59. The univariable and multivariable logistic regression analyses were performed to obtain the odds ratios (ORs) and the 95% confidence interval (CI) of each indicator in relation to inactivity during leisure time and inactivity during commuting. All *p* values were two-sided and *p* < 0.05 was considered as statistically significant. The whole statistical analysis was performed by the use of the STATISTICA Windows XP version 10.0 program (StatSoft Poland Inc., Tulusa, OK, USA).

## 3. Results

Three thousand six hundred and thirty six beneficiaries of social assistance were invited to take part in the study. The final analysis included 1817 SD people (response rate = 49.8%). The surveys with missing information were excluded. 

### 3.1. Baseline Characteristics of the Study Population

Baseline characteristics of the study population are presented in Table 1. In the group of 1817 participants, there were 1224 females and 593 males with the mean age of 39.2 ± 7.7 years (38.2 ± 7.2 for the females and 41.1 ± 8.1 years for the males; *p* < 0.001). Table 1 presents the prevalence of physical inactivity during leisure time and commuting among the women and men. Characteristics of the study population also include data on the education, employment status, subjective assessment of monthly income, health status and living conditions, number of health problems, alcohol consumption, smoking cigarettes, cohabitation with somebody or living alone and having children.

In the study, 27.7% of the respondents had primary education (the females—48.8% and the males—42.6%), 32.9% vocational education (the females—59.9% and the males—40.1%), 34% secondary education (the females—78.4% and the males—21.6%), and 5.4% higher education (the females—91.7% and the males—8.3%). The unemployed participants constituted a dominant group—58.7% (the females—74.8% and the males—25.2%). Almost one third of the study population had a permanent job (the females—58.2% and the males—41.8%), 8.6% declared having a temporary job, 3.0% were disabled or retired, and 0.1% were female students. According to the subjective assessment of monthly income, there were only 12% of the participants who answered that they had sufficient money to cover all living needs, and only 1.1% could save a certain amount. One half of the study population had monthly income which was sufficient to cover the basic needs only and one fourth of them had insufficient income to cover even the basic needs. Only 1.3% of the women and 0.7% of the men declared that their monthly income was sufficient to cover all their living needs plus to save a certain amount and 11.7% of the women and 9.3% of the men had monthly income to cover all their living needs. Fifty three point one percent of the women and 47.7% of the men declared that their monthly income was sufficient to cover the basic needs only, and 21.7% of the women and 32.7% of the men reported that their monthly income was insufficient to cover even the basic needs.

Further data presented in Table 1 show that the subjective health condition was considered as fair by 35.0% of the participants (35.6% among the females and 34.4% among the males), as rather fair by 31.0% of the respondents (33.6% among the females and 26.2% among the males), as neither fair nor poor by 23.3% (22.6% among the females and 24.7% among the males), as rather poor by 7.8% (6.6% among the females and 10.4% among the males) and as poor by 2.9% of them (2.0% among the females and 4.9% among the males). Majority of the respondents (66%) declared a good and a rather good health condition but health problems concerned 86.3% of the examined sample. Only 13.7% of the beneficiaries had no health problems (11.4%—the females and 18.4%—the males), 1–3 health problems were declared by 54.3% of the respondents (53.8%—the females and 55.1%—the males), 4–6 health problems concerned 26.3% of them (28.7%—the females and 21.3%—the males), while 7 and more health problems were reported by 5.7% (6.0%—the females and 5.1% the males).

With regard to the data on the use of alcohol and cigarette smoking, 46.9% of the study participants did not drink alcohol at all, while 53.1% drank moderately and heavily (46.8% of the females and 65.9% of the males). In the past, fewer social care beneficiaries smoked cigarettes—15.3%. In the past 14.5% of women smoked cigarettes, while 16.9% of men smoked cigarettes. Now, active smokers were 37.1% (29.6%—the females and 52.8%—the males). Forty seven point six percent of the study participants had never smoked cigarettes (the females—55.9% and the males—30.4%).

Forty six point eight percent of the respondents described subjective assessment of living conditions as fair and rather fair (the females—48.9% and the males—41.3%), 45.2% as neither fair nor poor (the females—42.7% and the males—49.2%), 4.9% as rather poor (the females—4.8%, the males—4.9%) and 1.8% described their living conditions as poor (the females—1.5% and the males—2.5%). Majority of the beneficiaries (84.5%) lived with a partner and/or a family (the females—84.6% and the males—84.5%) and most of them (67.5%) declared having children older than 15 years old (the females—67.8% and the males—66.6%). 

Obesity (BMI ≥ 30) concerned 23.8% of the participants (the females—23.6% and the males—24.3%).

### 3.2. Characteristic of the Prevalence of Physical Activity of (LTPA and CPA) among the Study Population

Characteristic of physical activity during leisure time and commuting among the beneficiaries is presented in Table 2. Three fourth of the study population (73.9%) did not meet the recommended levels of physical activity during leisure time (LTPA). More than one half (52.4%) of the respondents stated that they had none leisure-time physical activity, and the men (58.1%) declared it more often than the women (49.6%) (*p* < 0.05). There was also a statistically significant difference between the women (23.6%) who exercised occasionally more often than the men did (17.2%) (*p* < 0.05). Physical activity during leisure time was noted more rarely among the beneficiaries, i.e., 2–3 days weekly (9.4%) and 4–7 days weekly (16.7%), in the males—8.0% and 16.7%, respectively; and in the case of females—10.1% and 16.7%, respectively; (*p* > 0.05).

Over three fourth of the surveyed population (81.8%) did not practice commuting physical activity. Nine point six percent of the respondents declared spending 1–14 min per day (e.g., walking or cycling) on getting to work (the females—9.2% and the males—10.4%). Fewer participants (9.6%) practiced commuting physical activity for 15–29 min per day (the females—6.2% and the males—6.3%). The smallest group of the study participants practiced commuting for 30 min and more a day (2.4%) (the females—2.3% and the males—2.6%). There were no statistically significant differences between the males and females in any range of time needed to get to/from work in commuting PA (*p* > 0.05).

### 3.3. Correlates of Lack of LTPA and CPA

The results of the logistic regression analyses for inactivity during leisure time (LTPA) are presented in Table 3, for inactivity in commuting (CPA) in Table 4 and for both forms of physical inactivity (LTPA & CPA) in Table 5 by sociodemographic characteristics. The Odds Ratios (OR) and the 95% Confidence Intervals (CI) for inactivity during leisure time indicated that the men had much higher odds of leisure time physical inactivity compared to the women (OR = 1.35; 95% CI: 1.11–1.65; *p* ≤ 0.01). There was a statistically significant difference between the e-cigarettes users. Those who did not use e-cigarettes had much higher odds of leisure time physical inactivity compared to the e-cigarettes users (OR = 2.19; 95% CI: 1.12–4.25; *p* ≤ 0.05).

Physical inactivity during commuting looked different. The Odds Ratios (OR) and the 95% Confidence Intervals (CI) for commuting inactivity showed that the level of education played a significant role. The study participants with primary, vocational and secondary education had higher odds of commuting inactivity than the participants with a high level education (for the primary education OR = 0.34; 95% CI: 0.18–0.64; *p* ≤ 0.001; for vocational education OR = 0.44; 95% CI: 0.24–0.80; *p* ≤ 0.01 and for secondary education OR = 0.54; 95% CI: 0.29–0.99; *p* ≤ 0.05). The unemployed study participants had much higher odds of inactivity CPA than those beneficiaries who had a permanent or a temporary job (OR = 3.41; 95% CI: 2.59–4.48; *p* ≤ 0.05). Also the respondents who consumed alcohol had higher odds of inactivity CPA than those who did not drink alcohol (OR = 1.86; 95% CI: 1.43–2.41; *p* ≤ 0.001).

The Odds Ratios (OR) and the 95% Confidence Intervals (CI) for inactivity during leisure time (LTPA) and during commuting (CPA) together, once again, showed that the men had much higher odds of physical inactivity during leisure time (LTPA) and commuting (CPA) compared to physical inactivity of the women (OR = 1.22; 95% CI: 1.00–1.50; *p* ≤ 0.05).

### 3.4. Reasons for Not Taking Up Physical Activity among the Study Population

Reasons for physical inactivity among the beneficiaries are presented in Table 6.

The most common reason why participants did not take up physical activity was high general physical activity (e.g., physical work, a house, a garden plot etc.). This particular reason referred to 36.4% of the examined sample (36.4%—the females and 36.5% of the males). There was not a statistically significant difference between the women and men (*p* > 0.97). The further two important reasons why the beneficiaries did not exercise were: lack of time—28.1% (the females—30.8% and 23.2%—the males) and no willingness to exercise—25.4% (27.0%—the females and 22.6%—the males). The women declared lack of time to exercise more frequently than the men did and the difference was statistically significant (*p* < 0.017). Other reasons were: bad health condition (a disease, disability)—12.2% of the study group (the females—8.7% and the males—18.4%), lack of money—3.5% (the females—3.6% and 3.2% the males), and no access to suitable facilities (a gym, playing field etc.)—2.4% (the females—2.5%, and the males—2.3%).

## 4. Discussion

The present study examined the prevalence of physical activity (LTPA and CPA) among the adult beneficiaries of government welfare assistance in Poland. It also collected data on the reasons of inactivity among the studied population. The results showed that 52.4% of the participants had none physical activity during leisure time and 81.8% had no physical activity during commuting. Comparing these results to the general population of Poland, it appears that the prevalence of physical inactivity (LTPA) among the beneficiaries of welfare group is 1.5 times higher than in the general population [41]. In addition, three fourth of the study participants did not reach the physical activity recommendation level in LPTA (73.9%). What is more, four fifth of the participants also did not meet the recommended level of commuting CPA (91.4%). Both cases of physical inactivity (LTPA & CPA) were also higher when compared to the general population [41].

Only 16.7% of the respondents exercised between 4–7 times weekly during their leisure time (LTPA). The prevalence of regular physical activity (more days weekly) among the beneficiaries was twice time lower than it was among adults in the general population of Poles [41]. 

On the other hand, the prevalence of active commuting (CPA) lasting all the studied time periods (<15 min, 15–30 min and >30 min and more) was several times lower among the beneficiaries than it was in the general population [12,41].

In studied group, the men did not practice LTPA more frequently when compared to the women, contrary to the situation in the general population of Poland, where inactivity is more common among women than men [41]. However, in the case of inactivity during commuting (CPA), in the socially disadvantaged group higher inactivity was reported among the women than in the men. The same has been observed in the general population of Poland [11,41].

A study conducted by Marques A. et al., in 28 European countries among adults (18–65 years) has shown that physical activity recommended levels were attained by 64.5% of the study participants, and similarly to the general Polish population, women exercised more often than men did (66.49% vs. 63.36%) [17]. Although research in several countries (e.g., Portugal, The Netherlands, Luxembourg, France, Belgium) has shown a slightly higher physical activity among women than men; in our study the situation was opposite. It could be associated with the fact that the socially disadvantaged women had more workload at home and in a different way shared the time devoted to responsibilities. Further data of our study indicated that the socially disadvantaged women declared not taking up PA because of lack of time. In our study, age, subjective assessment of living conditions, subjective assessment of monthly income and of health status as well as cohabitation with a partner and/or family, having children, obesity as well as smoking status were factors, which were not correlated with physical inactivity during leisure-time and commuting among the adult beneficiaries of government welfare assistance in Poland.

The multivariable analyses indicated only four significant factors correlated with physical inactivity. During leisure-time (LTPA) physical inactivity correlated positively with male gender, and during commuting PA (CPA) with the level of education, employment status, health problems and alcohol consumption. The men had a 1.3 higher risk of not doing physical exercises than the women and, they declared a bad health condition more often than the lack of time (*p* < 0.05). In the general population of Poland, men suffer from non-communicable diseases more frequently than women. Physical inactivity constitutes a serious risk factor for the disadvantaged men to develop non-communicable diseases earlier and more frequently than the women. The socially disadvantaged groups are unaware of consequences of physical inactivity because of lack of knowledge and not understanding the risk of developing a coronary heart disease [42].

Another significant correlate of physical inactivity during commuting PA (CPA), which was indicated by the multivariable analyses, was the level of education. The individuals with a higher level of education were statistically least likely to use CPA when compared to the people with a secondary (OR = 0.54; 95% Cl 0.29–0.99 *p* < 0.05), vocational (OR = 0.44; 95% Cl 0.24–0.80 *p* < 0.01) and a primary level of education (OR = 0.34; 95% Cl 0.18–0.64 *p* < 0.001). We can only suppose that in our study it was associated with the presence of more than one vehicle in a household, similarly to the studies conducted among adult Brazilians and people living in six middle income countries, where the vehicles constituted a reason both in the case of men and women for being physically inactive during commuting [22,43].

It is surprising as it seems that highly educated people should have knowledge on health benefits of physical activity [44]. In our study the highly educated individuals did not exercise in leisure time more often than the persons with lower education (*p* > 0.05). In the study described in the paper, commuting physical activity (CPA) was negatively associated with being unemployed (odds ratio even free times higher—OR = 3.41; 95% Cl 2.59–4.48 *p* < 0.05) [17] and being a moderate and heavy drinker of alcohol (odds ratio almost twice times higher—OR = 1.86; 95% Cl 1.43–2. *p* < 0.001). This is in agreement with results of the study performed among Canadians, which has shown that the adults who drank alcohol were less physically active [45].

The univariable logistic regression showed that the disadvantaged people who had health problems (1–3 problems OR = 1.38; 95% Cl 1.01–1.90 *p* < 0.05, and 4–6 problems OR = 1.61; 95% Cl 1.12–2.32 *p* < 0.01) were less likely to be active when compared to the healthy individuals. A meta-analysis by O’Mara-Eves A. et al has shown that a worse health condition in disadvantaged groups was observed in many developed countries, where they had worse health outcomes such as lower life expectancy than non-disadvantaged groups [46]. After an effective commuting intervention they successfully managed to increase self-efficacy of disadvantaged group in terms of health consequences and health behavior [46]. It is worth to use both forms of physical activity, i.e., LTPA and CPA, as they are both extremely important with respect to the primary and secondary prevention of CVD. Moreover, they make it easier to deal with daily tasks (such as climbing stairs and shopping), they improve well-being and contribute considerably to an increase in life expectancy [47,48]. Finally, one more of the most important findings of the study was getting to know the reasons why the adult beneficiaries of government welfare assistance in Poland are physically inactive. The most often declared reason for physical inactivity was a high general physical activity (physical work, a house, a garden plot etc.), both among the men and women (*p* > 0.05). Then, there were: lack of time (31% of the females vs. 23.2% of the men, *p* < 0.05), no willingness to exercise (27% of the females vs. 22.6% of the men, *p* > 0.05) and bad health condition (a disease, disability—8.7% of the females vs. 18.4% of the men, *p* < 0.05). Only 3.5% of the studied disadvantaged group declared lack of money as a reason for physical inactivity and 2.4% of them mentioned access to suitable facilities (a gym, playing field etc.). There were no statistical differences between the female and male participants of the study, both in the case of lack of money and access to facilities (*p* > 0.05). Lack of time for PA (and LTPA) is characteristic not only for the disadvantaged population. Results of the reasons for physical inactivity are similar in general Polish population and related to lack of time (26.7%), poor health (24.3%) and no willingness/interest to exercise (24%) [42]. Also in the case of people with higher education and a higher occupation status, being sufficiently physically active during their leisure time was a significant problem [44]. However, in the case of the socially disadvantaged group, physical activity can result in higher self-efficacy than necessary to overcome daily difficulties [44]. 

Recommendations in systematic reviews, where the amount, frequency, intensity and a type of physical activity have been examined, required to achieve physical and mental health benefits in the adult population, consistently indicate a weekly volume of 150 min of moderate intensity physical activity. The most significant thing is that this overall volume of physical activity is more important than the specific type of activity, its intensity or frequency of sessions. For example: gardening is a great way to enjoy some fresh air, it is fun, creative and helps to manage stress. However, gardening is hardly pumping iron and it probably won’t do much good for you in terms of cardiovascular fitness. On the other hand, digging, planting, weeding, and other repetitive tasks that require strength or stretching are excellent forms of a low-impact exercise (can help strengthen bones and joints), especially in the case of people who find more vigorous exercise a challenge, such as those who are older, have disabilities or those who suffer from chronic pain [49,50,51]. Different types of housework constitute another example: ironing, cleaning and dusting (performed by women more frequently). They are of light intensity and are associated with energy expenditure (hoovering moderate intensity). Nevertheless, they are boring, monotonous, static, tiring and often involve only some groups of muscles, which results from a forced body position and they do not result in the desired health effects. What is more, they are often performed due to such a necessity and not because people like doing them, which is the most important thing when it comes to improving health by being physically active [49,50,51]. 

Humans are not programmed for a sedentary lifestyle. Physical activity has well-documented health benefits in the primary, secondary and tertiary prevention of non-communicable diseases, and it is recommended as a factor improving prognosis. Cohort studies have proved the fact that a high level of leisure time physical activity (LTPA) results in a significant reduction in the prevalence of both coronary heart disease and stroke in both sexes [2]. The adult beneficiaries of government welfare assistance should have this basic knowledge in order to be more motivated to do physical exercises and to experience numerous health benefits of it. 

## 5. Strengths and Limitations of the Study

The current analysis has several strengths. Firstly, the study was conducted among the socially disadvantaged population of social assistance beneficiaries from a rural district of Poland. Secondly, a large group of beneficiaries aged 18–59 (3636 persons) were invited to take part in the study. Finally, one half of the invited group participated in the survey. The response rate was almost 50% (response rate = 49.8%) of the average level compared to other questionnaire surveys conducted in Poland. Another important strength of the study is the fact that it examined the prevalence of physical activity among the beneficiaries of government welfare assistance, both during leisure time and during commuting. It is the study where for the first time both types of physical activity have been examined with regard to this characteristic group in Poland. In general, there are not many studies that have examined both types of physical activity in a disadvantaged population, not to mention studies conducted in Poland. The fact that the study examined a number of determinants that can influence physical inactivity among the study population is its yet another strength. Finally, the study results will be of great importance when developing physical activity programs addressed to this special group (and not to the general population), which will most probably translate into their efficiency and success. 

Limitations of the study include the fact that it examined the participants only at a single point in time, i.e., now. We know nothing about physical activity among the examined beneficiaries in the past, e.g., practiced sports, education in school and home environment, which could have determined their physical activity in the future. In order to result in health benefits, physical intervention should be long-term and it cannot only cover one or several months. It is difficult to start regular exercise but it is even more difficult to maintain physical activity for years. Another limitation of the study refers to the questions used in the survey. The questionnaire consisted of several parts of questions but all of them were direct questions, which required one answer from the participants or had to be missed out. More open questions could help to find out about the needs and barriers that this group of respondents has. Self-reporting nature of the process of data collection is associated with the evident risk of recall bias and this is also a limitation of the study that has to be mentioned.

## 6. Conclusions

The prevalence of physical inactivity (both LTPA and CPA) among the social assistance recipients is much higher than in the general population. Therefore, especially rural disadvantaged residents are undoubtedly an appropriate target group for future physical activity interventions. Several factors significantly correlate with the physical inactivity in this socio-demographic group. When promoting an active lifestyle (PA leisure-time and/or commuting) and prevention of non-communicable diseases we should remember about these factors and pay attention to substantial differences between the general population and the socially disadvantaged individuals. Improved continued monitoring of physical activity is required. Furthermore, continuing surveillance among general and vulnerable populations is necessary to evaluate motivators and barriers towards active living.

## Figures and Tables

**Table 1:** Characteristics of the study population (n = 1817).

Variable	Total Sample	Women				Men			
		Total	Inactive LTPA	Inactive CPA	Inactive LTPA and CPA	Total	Inactive LTPA	Inactive CPA	Inactive LTPA and CPA
Overall	n = 1817 n (%)	n = 1224 n (%)	n = 556 (45.5%) n (%)	n = 996 (81.4%) n (%)	n = 485 (39.6%) n (%)	n = 593 n (%)	n = 311 (52.4%) n (%)	n = 473 (79.8%) n (%)	n = 267 (45%) n (%)
**Age (years)**									
**18–29**	209(11.5)	160 (76.6)	77 (48.1)	137 (85.6)	72 (45.0)	49 (23.4)	22 (44.9)	38 (77.6)	20 (40.8)
**30–39**	778 (42.8)	566 (72.8)	253 (44.7)	467 (82.5)	221 (39.0)	212 (27.2)	120 (56.6)	170 (80.2)	104 (49.0)
**40–49**	601 (33.1)	385 (64.1)	177 (46.0)	304 (79.0)	148 (38.4)	216 (35.9)	116 (53.7)	174 (80.6)	97 (44.9)
**50–59**	229 (12.6)	113 (49.3)	49(43.4)	88 (77.9)	44 (38.9)	116 (50.7)	53 (45.7)	91 (78.4)	46 (39.7)
**Education**									
**Primary**	493 (27.7)	283 (57.4)	138 (48.8)	162 (57.2)	117 (41.3)	210 (42.6)	103 (49.0)	162 (77.1)	86 (40.9)
**Vocational**	586 (32.9)	351 (59.9)	151 (43.0)	192 (54.7)	132 (37.6)	235 (40.1)	132 (56.2)	192 (81.7)	115 (48.9)
**Secondary**	606 (34.0)	475 (78.4)	215 (45.3)	106 (22.3)	190 (40.0)	131 (21.6)	67 (51.1)	106 (80.9)	57 (43.5)
**High**	96 (5.4)	88 (91.7)	36 (40.9)	6 (6.8)	34 (38.6)	8 (8.3)	4 (50.0)	6 (75.0)	4 (50.0)
**Missing data**	36 (2.0)	27 (2.2)	16 (2.9)	7 (0.7)	12 (2.5)	9 (1.5)	1 (0.3)	7 (1.5)	5 (1.9)
**Employment status**									
**Permanent job**	533 (29.5)	310 (58.2)	150 (4.8)	220 (71.0)	116 (21.8)	223 (41.8)	128 (57.4)	178 (79.8)	108 (48.4)
**Temporary job**	156 (8.6)	85 (54.5)	37 (43.5)	54 (63.5)	28 (32.9)	71 (45.5)	32 (45.1)	38 (54.5)	21 (29.6)
**Disabled or retired**	54 (3.0)	26 (48.1)	8 (30.8)	21 (80.9)	6 (23.1)	28 (51.9)	14 (50.0)	19 (67.9)	13 (46.4)
**Student**	2 (0.1)	2 (100.0)	0 (0.0)	1 (50.0)	0 (0.0)	0 (0.0)	0 (0.0)	0 (0.0)	0 (0.0)
**Unemployed**	1060 (58.7)	793 (74.8)	356 (44.9)	693 (87.4)	331(41.4)	267 (25.2)	135 (50.6)	236 (88.4)	123 (46.1)
**Missing data**	12 (0.7)	8 (0.7)	5 (0.9)	7 (0.7)	4 (0.8)	4 (0.7)	2 (0.6)	2 (0.4)	2 (0.7)
**Subjective assessment of monthly income**									
**Sufficient to cover all living needs plus may save a certain amount**	20 (1.1)	16 (80.0)	2 (12.5)	14 (87.5)	2(12.5)	4(20.0	2(50.0)	4(100.0)	2(50.0)
**Sufficient to cover all living needs**	198 (10.9)	143 (72.2)	26 (18.2)	117 (81.8)	59 (41.2)	55 (27.8)	39 (70.1)	45 (81.8)	33 (60.0)
**Sufficient to cover the basic living needs only**	933 (51.5)	650 (69.7)	318 (48.9)	546 (84.0)	283 (43.5)	283 (30.3)	159 (56.2)	224 (79.1)	133 (47.0)
**Not sufficient to cover even the basic living needs**	454 (25.1)	266 (58.6)	115 (43.2)	206 (77.4)	97 (36.5)	188 (41.4)	85 (45.2)	151 (80.3)	76 (40.4)
**Declined response difficult to say**	141 (7.8)	101 (71.6)	32 (31.7)	70 (69.3)	26 (25.7)	40 (28.4)	16 (40.0)	31 (77.5)	15 (37.5)
**Missing data**	71 (3.9)	48 (3.9)	22 (3.9)	37 (3.7)	18 (3.7)	23 (3.9)	10 (3.2)	18 (3.8)	8 (3.0)
**Subjective health state**									
**Fair**	632 (35.0)	434 (35.6)	184 (42.4)	341 (78.6)	156 (35.9)	198 (34.3)	106 (53.5)	154 (77.8)	86 (43.4)
**Rather fair**	559 (31.0)	405 (33.6)	186 (45.9)	333 (82.2)	157 (38.8)	154 (26.2)	85 (55.2)	118 (76.6)	70 (45.5)
**Neither fair nor poor**	420 (23.3)	275 (22.6)	135 (49.1)	231 (84.0)	126 (45.8)	145 (24.7)	66 (42.8)	119 (82.1)	61 (42.1)
**Rather poor**	141 (7.8)	80 (6.6)	36 (45.0)	64 (80.0)	33 (41.2)	61 (10.4)	34 (55.7)	50 (82.0)	32 (52.4)
**Poor**	53 (2.9)	24 (2.0)	11 (45.8)	22 (91.7)	10 (41.7)	29 (4.9)	17 (58.6)	26 (89.6)	15 (51.2)
**Missing data**	12 (0.7)	6 (0.5)	4 (0.7)	5 (0.5)	3 (0.6)	6 (1.0)	3 (1.0)	6 (1.3)	3 (1.1)
**Health problems**									
**None**	245 (13.7)	137 (11.4)	62 (45.2)	108 (78.8)	54 (39.4)	108 (18.4)	62 (57.4)	78 (72.2)	48 (44.4)
**1–3 health problems**	968 (54.3)	645 (53.8)	276 (42.8)	519 (80.5)	238 (36.9)	323 (55.1)	170 (52.6)	266 (82.3)	150 (464)
**4–6 health problems**	469 (26.3)	344 (28.7)	177 (51.4)	292 (86.0)	160 (46.5)	125 (21.3)	57 (45.6)	99 (79.2)	51 (40.8)
**≥7 health problems**	102 (5.7)	72 (6.0)	32 (44.4)	59 (81.9)	25 (34.7)	30 (5.1)	16 (53.3)	23 (76.6)	12 (40.0)
**Missing data**	33 (1.8)	26 (2.1)	9 (1.6)	18 (1.8)	8 (1.6)	7 (1.2)	6 (1.9)	7 (1.5)	6 (2.2)
**Alcohol consumption**									
**Does not drink at all**	802 (46.9)	619 (59.2)	287 (46.8)	481 (78.4)	246 (40.1)	189 (34.1)	100 (52.9)	144 (76.2)	81(42.9)
**Moderate drinking and heavy drinking**	905 (53.1)	540 (46.8)	247 (45.7)	460 (85.2)	222 (41.1)	365 (65.9)	202 (55.3)	301 (82.5)	178 (48.8)
**Missing data**	110 (6.0)	71 (5.8)	22 (4.0)	55 (5.5)	17 (3.5)	39 (8.6)	9 (2.9)	28 (5.9)	8 (3.0)
**Smoking cigarettes**									
**Past use**	278 (15.3)	178 (64.0)	85 (47.7)	143 (80.3)	74 (41.6)	100 (36.0)	48 (48.0)	85 (85.0)	45 (45.0)
**Current use**	675 (37.1)	362 (53.6)	166 (45.8)	295 (81.5)	146 (40.3)	313 (46.4)	160 (51.1)	242 (77.3)	132 (42.2)
**Never use**	864 (47.6)	684 (79.2)	305 (44.6)	558 (81.6)	265 (38.7)	180 (20.8)	103 (57.2)	146 (81.8)	90 (50.0)
**Missing data**	0 (0.0)	0 (0.0)	0 (0.0)	0 (0.0)	0 (0.0)	0 (0.0)	0 (0.0)	0 (0.0)	0 (0.0)
**Subjective assessment of living conditions**									
**Fair**	194 (10.8)	132 (68.0)	53 (40.2)	101 (76.5)	44 (33.3)	62 (32.0)	35 (56.5)	45 (72.6)	26 (41.9)
**Rather fair**	650 (360)	467 (71.8)	225 (48.2)	392 (83.9)	199 (42.6)	183 (28.2)	102 (55.7)	156 (85.2)	92 (50.3)
**Neither fair nor poor**	815 (45.2)	523 (64.2)	226 (43.2)	422 (80.7)	197 (37.7)	292 (35.8)	152 (52.0)	226 (77.4)	131 (44.9)
**Rather poor**	88 (4.9)	59 (67.0)	29 (49.2)	44 (74.6)	24 (40.7)	29 (33.0)	12 (41.4)	22 (75.9)	9 (31.0)
**Poor**	33 (1.3)	18 (54.5)	11 (61.1)	17 (94.4)	11 (61.1)	15 (45.5)	6 (40.0)	14 (93.3)	6 (40.0)
**Declined response difficult to say**	23 (1.3)	16 (69.6)	7 (43.7)	15 (93.7)	7 (43.7)	7 (30.4)	2 (28.6)	7 (100.0)	2 (28.6)
**Missing data**	14 (0.8)	9 (0.7)	5 (0.9)	5 (0.5)	3 (0.6)	5 (0.8)	2 (0.6)	3 (0.6)	1 (0.4)
**Cohabitation with a partner and/or family**									
**No (living alone)**	281 (15.5)	189 (67.2)	73 (38.6)	153 (80.9)	58 (30.7)	92 (32.8)	54 (58.7)	75 (81.5)	47 (51.1)
**Yes**	1536(84.5)	1035(67.4)	483 (46.7)	843 (81.4)	427 (41.3)	501 (32.6)	257 (51.3)	398 (79.4)	220 (43.9)
**Children < 15 years**									
**Yes**	1226 (67.5)	831 (67.8)	402 (48.4)	675 (81.2)	169 (20.3)	395 (32.2)	198 (50.1)	314 (79.5)	352 (89.1)
**No**	591 (32.5)	393 (66.5)	154 (39.2)	321 (81.7)	98 (24.9)	198 (33.5)	113 (57.1)	159 (80.3)	133 (67.2)
**Obesity**									
**Yes (≥30)**	433 (23.8)	289 (66.7)	134 (46.4)	232 (80.3)	115 (39.8)	144 (33.3)	74 (51.4)	109 (75.7)	60 (41.7)
**No (<30)**	1384 (76.2)	935 (67.6)	422 (45.1)	764 (81.7)	370 (39.6)	449 (32.4)	237 (52.8)	364 (81.1)	207 (46.1)

LTPA, leisure time physical activity; CPA, commuting physical activity.

**Table 2:** Physical activity characteristics of the study sample (*n* = 1817).

Physical Activity	Total	%	Women		Men		*p*
	n		n	% (95% CI)	n	% (95% CI)	
**Leisure time**							
None	867	52.4	536	49.6 (46.7–52.5)	311	58.1 (53.9–62.3)	*p* < 0.05
Occasionally	357	21.5	265	23.6 (21.1–26.1)	92	17.2 (14.0–20.4)	*p* < 0.05
2–3/week	156	9.4	113	10.1 (8.3–11.7)	43	8.0 (5.7–10.3)	*p* > 0.05
4–7/week	276	16.7	187	16.7 (14.5–18.9)	89	16.7 (13.5-19.9)	*p*> 0.05
Missing data	161	8.9	103	8.4	58	9.8	
**Commuting**							
None	1469	81.8	996	82.3 (80.2–84.4)	473	80.7 (77.5–83.9)	*p* > 0.05
1–14 min; (walking or cycling) per day	172	9.6	111	9.2 (7.6–10.8)	61	10.4 (7.9–12.9)	*p* > 0.05
15–29 min; (walking or cycling) per day	112	6.2	75	6.2 (4.8–7.6)	37	6.3 (4.3–8.3)	*p* > 0.05
30 min and more; (walking or cycling) per day	43	2.4	28	2.3 (1.5–3.1)	13	2.6 (1.3–3.9)	*p* > 0.05
Missing data	21	1.2	14	1.1	7	1.2	

**Table 3:** Odds ratio (OR) and 95% confidence interval (CI) for inactivity during leisure time by socio-demographic characteristics.

Characteristic	Total n = 867				
		Univariable Logistic Regression		Multivariable Logistic Regression	
	N %	OR	95% CI	OR	95% CI
**Gender**					
Female	556 (45.4)	1.00	reference	1.00	reference
Male	311 (52.5)	1.32	1.09–1.61 **	1.35 **	1.11–1.65
**Smoking status**					
Smoker	326 (48.3)	1.04	0.85–1.27		
Non-smoker	541 (47.4)	1.00	reference	1.00	reference
**Age (years)**					
<30	99 (47.4)	1.00	reference		
30–39	373 (47.9)	1.02	0.72–1.44		
40–49	293 (48.7)	1.06	0.74–1.51		
50–59	102 (44.5)	0.89	0.60–1.33		
**Subjective assessment of living condition**					
Good	415 (49.2)	1.00	reference		
Less than good	445 (46.4)	0.89	0.74–1.09		
**Education**					
Primary	241 (48.8)	1.11	0.76–1.64		
Vocational	283 (48.3)	1.09	0.74–1.59		
Secondary	282 (46.5)	1.04	0.69–1.48		
High	40 (41.8)	1.00	reference		
**Employment status**					
Permanent or temporary job	347 (50.4)	1.00	reference		
Disabled or retired	22 (40.7)	0.67	0.38–1.18		
Unemployed	491 (46.2)	0.84	0.70–1.02		
**Subjective assessment of monthly income**					
Good	110 (50.5)	1.00	reference		
Less than good	725 (47.5)	0.89	0.67–1.18		
**Subjective health state**					
Good	561 (47.1)	1.00	reference		
Less than good	299 (48.7)	1.07	0.88–1.30		
**Health problems**					
None	124 (50.6)	1.00	reference		
1–3 health problems	443 (46.0)	0.85	0.65–1.11		
4–6 health problems	234 (49.9)	1.00	0.74–1.34		
≥7 health problems	48 (47.1)	0.89	0.56–1.40		
**Cont.**					
**Alcohol consumption**					
Does not drink at all	387 (48.2)	1.00	reference		
Moderate drinking and heavy drinking	449 (49.6)	1.06	0.87–1.28		
**Cohabitation with a partner and/or family**					
No (living alone)	127 (45.2)	1.00	reference		
Yes	740 (48.2)	1.13	0.88–1.45		
**Children < 15 years**					
Yes	600 (48.9)	1.00	reference		
No	267 (45.2)	0.86	0.71–1.05		
**Obesity**					
Yes (≥30)	208 (48.0)	1.02	0.82–1.26		
No (<30)	659 (47.6)	1.00	reference		

Fully adjusted model including all the statistically significant characteristics. ** *p* ≤ 0.01.

**Table 4:** Odds ratio (OR) and 95% confidence interval (CI) for inactivity during commuting by socio-demographic characteristics.

Characteristic	Total n = 1469				
		Univariable Logistic Regression		Multivariable Logistic Regression	
	N %	OR	95% CI	OR	95% CI
**Gender**					
Female	996 (81.4)	1.00	reference		
Male	473 (79.8)	0.90	0.70–1.15		
**Smoking status**					
Smoker	537 (79.6)	0.88	0.69–1.11		
Non-smoker	932 (81.6)	1.00	reference		
**Age (years)**					
<30	175 (83.7)	1.00	reference		
30–39	637 (81.9)	0.88	0.58–1.32		
40–49	478 (79.5)	0.76	0.50–1.15		
50–59	179 (78.2)	0.70	0.43–1.13		
**Subjective assessment of living condition**					
Good	694 (82.2)	1.00	reference		
Less than good	767 (80.0)	0.86	0.68–1.09		
**Education**					
Primary	27 (75.0)	0.49 **	0.27–0.86	0.34 ***	0.18–0.64
Vocational	463 (79.0)	0.48 **	0.27–0.86	0.44 **	0.24–0.80
Secondary	500 (82.5)	0.60	0.34–1.08	0.54 *	0.29–0.99
High	90 (93.7)	1.00	reference	1.00	reference
**Employment status**					
Permanent or temporary job	490 (71.1)	1.00	reference	1.00	reference
Disabled or retired	40 (74.1)	1.15	0.61–2.17	1.43	0.71–2.87
Unemployed	930 (87.6)	2.85 ***	2.23–3.64	3.41 ***	2.59–4.48
**Subjective assessment of monthly income**					
Good	186 (85.3)	1.00	reference		
Less than good	1228 (80.4)	0.70	0.47–1.05		
**Subjective health state**					
Good	946 (79.4)	1.00	reference	1.00	reference
Less than good	512 (83.4)	1.30*	1.01–1.68	1.35	0.99–1.84
**Health problems**					
None	186 (75.9)	1.00	reference	1.00	reference
1–3 health problems	781 (81.2)	1.38 *	1.01–1.90	1.37	0.97–1.93
4–6 health problems	391 (83.4)	1.61 **	1.12–2.32	1.33	0.88–2.02
≥7 health problems	82 (80.4)	1.32	0.75–2.30	1.03	0.54–1.94
**Alcohol consumption**					
Does not drink at all	625 (77.9)	1.00	reference	1.00	reference
Moderate drinking and Heavy drinking	761 (84.1)	1.49 ***	1.17–1.91	1.86 ***	1.43–2.41
**Cohabitation with a partner and/or family**					
No (living alone)	228 (81.3)	1.00	reference	1.00	reference
Yes	1241 (84.1)	0.98	0.73–1.32		
**Children <15 years**					
Yes	989 (80.7)	1.00	reference		
No	480 (81.2)	1.03	0.80–1.35		
**Obesity**					
Yes (≥30)	341 (78.7)	0.84	0.64–1.10		
No (<30)	1128 (81.5)	1.00	reference		

Fully adjusted model including all the statistically significant characteristics. *p ≤ 0.05. ** *p* ≤ 0.01. *** *p* ≤ 0.001.

**Table 5:** Odds ratio (OR) and 95% confidence interval (CI) for inactivity during leisure time and during commuting by socio-demographic characteristics.

Characteristic	Total (n = 1817)				
		Univariable Logistic Regression		Multivariable Logistic Regression	
	N %	OR	95% CI	OR	95% CI
**Gender**					
Female	485 (39.6)	1.00	reference	1.00	reference
Male	267 (45.0)	1.25 *	1.02–1.52	1.22 *	1.00–1.50
**Smoking status**					
Smoker	278 (41.2)	0.99	0.81–1.20		
Non-smoker	478 (41.5)	1.00	reference	1.00	reference
**Age (years)**					
<30	92 (44.0)	1.00	reference		
30–39	325 (4.8)	0.91	0.67–1.24		
40–49	245 (40.8)	0.88	0.64–1.20		
50–59	90 (39.3)	0.82	0.56–1.21		
**Subjective assessment of living condition**					
Good	361 (42.8)	1.00	reference		
Less than good	387 (40.4)	0.91	0.75–1.09		
**Education**					
Primary	203 (41.2)	1.01	0.66–1.45		
Vocational	247 (42.2)	1.08	0.69–1.50		
Secondary	247 (40.8)	1.01	0.66–1.41		
High	38 (39.6)	1.00	reference		
**Employment status**					
Permanent or temporary job	273 (39.6)	1.00	reference		
Disabled or retired	19 (35.2)	0.82	0.46–1.47		
Unemployed	454 (42.8)	1.13	0.93–1.37		
**Subjective assessment of monthly income**					
Good	96 (44.0)	1.00	reference		
Less than good	630 (41.2)	0.89	0.67–1.19		
**Subjective health state**					
Good	469 (39.4)	1.00	reference	1.00	reference
Less than good	277 (45.1)	1.27 *	1.04–1.54	1.24 *	1.02–1.52
**Health problems**					
None	102 (41.6)	1.00	reference		
1–3 health problems	386 (40.1)	0.94	0.72–1.23		
4–6 health problems	211 (45.0)	1.15	0.85–1.55		
≥7 health problems	37 (36.3)	0.80	0.50–1.29		
**Alcohol consumption**					
Does not drink at all	327 (40.8)	1.00	reference		
Moderate drinking and heavy drinking	400 (44.2)	1.15	0.95–1.39		
**Cohabitation with a partner and/or family**					
No (living alone)	105 (37.4)	1.00	reference		
Yes	647 (42.1)	1.22	0.94–1.58		
**Children < 15 years**					
Yes	521 (42.5)	1.00	reference		
No	231 (39.1)	0.87	0.71–1.06		
**Obesity**					
Yes (≥30)	175 (40.4)	0.95	0.76–1.18		
No (<30)	588 (41.7)	1.00	reference		

Fully adjusted model including all the statistically significant characteristics. * *p* ≤ 0.05.

**Table 6:** Reasons for not taking up leisure time physical activity (LTPA) by the examined participants.

Reasons	Total n = 862 n (%)	Women n = 552 n (%)	Men n = 311 n (%)	*p*
Lack of time	242 (28.1)	170 (30.8)	72 (23.2)	0.0172
No willingness to exercise (I have no such a need)	219 (25.4)	149 (27.0)	70 (22.6)	0.1545
Bad health condition (a disease, disability)	105 (12.2)	48 (8.7)	57 (18.4)	0.0000
High general PA (physical work, a house, a garden plot etc.)	314 (36.4)	201 (36.4)	113 (36.5)	0.9766
Lack of money	30 (3.5)	20 (3.6)	10 (3.2)	0.7577
No access to suitable facilities (a gym, playing field etc.)	21 (2.4)	14 (2.5)	7 (2.3)	0.8547
Other reasons	1 (0.1)	1 (0.2)	0 (0.0)	0.4308
Missing data	5 (0.6)	4 (0.7)	1 (0.3)	-

## Supplementary Materials

The following are available online at www.mdpi.com/1660-4601/14/10/1126/s1.

## Abbreviations

PA	physical activity
LTPA	leisure time physical activity
CPA	commuting physical activity
